# Essentials of Human Physiology

**Published:** 1905-12

**Authors:** 


**Essentials of Human Physiology.** D. Noël Paton, M.D. Second Edition. Pp. xiii., 444. Edinburgh: Wm. Green & Sons. 1905.

This excellent text-book is probably better known in Scotland and the North of England than in the south. Its special features are that it is intended to deal with those parts of the now gigantic subject of physiology that are likely to be of practical importance to the medical student, and purely theoretical and scientific considerations are relegated to mere notices or altogether omitted. It is, moreover, intended to be read side by side with two specified books on practical physiology.

As illustrations of this method, it may be mentioned that the author describes how to feel a pulse and estimate tension, etc., and gives explanations of the sounds heard on auscultating the chest in full, even referring to the different types of cardiac bruits. He discusses the alleged hypersecretion of thyroid juice in exophthalmic goitre, and goes into details as to the reaction of degeneration in man, but on the other hand he does not so much as mention the various theories of hearing, discusses those of colour perception in about a dozen lines, barely touches on the mechanism of lymph production and the experimental data on which modern views depend, omits to mention acromegaly when discussing the pituitary body, and attempts to crowd Ehrlich's theory of side chains and immunity, with a reference also to toxins, cytotoxins, and precipitins, into two pages of large print.

There will probably be some difference of opinion as to the desirability of this method, depending on the larger question whether we should look upon physiology as a science *per se*, or as a mere stepping-stone to medicine. In these days of over-burdened medical curricula the latter view will certainly find supporters, and they will do well to recommend this book. It will probably be quite adequate for the Conjoint Board, but is certainly insufficient for the Intermediate M.B. Lond. or the First F.R.C.S. Eng. It is written in the "summary" style, and every sentence is important.

Dr. Paton has met with praiseworthy success in endeavouring to bring the book up to date—a most difficult accomplishment in modern physiology. There are a few points on which there would now be difference of opinion; it is now thought that the coronary arteries fill in systole, that the vaso-dilator nerves leave the cord in the posterior nerve roots, and we have got beyond the days of the stromuhr for measuring the velocity of the blood, Dr. Stewart's investigations on which are not mentioned.

The printing is beautifully clear, and the book does not take long to read through. The illustrations are very diagrammatic. One or two of them, *e.g.* the obsolete one on page 180, might be omitted.

